# A comparative analysis of outcomes in patients with aneurysmal subarachnoid hemorrhage based on surgical timing

**DOI:** 10.3389/fsurg.2025.1682278

**Published:** 2025-11-21

**Authors:** Meixue Wang, Jianming Zhai, Liping Liao, Chunying Cui, Liangzhen Zhu, Qingjian Wu

**Affiliations:** 1 Clinical Medical College of Jining Medical University, Jining, China; 2 Department of Emergency, Jining No. 1 People’s Hospital, Jining, China; 3 Department of Neurology, The Second Affiliated Hospital of Shandong First Medical University & Shandong Academy of Medical Sciences, Jinan, China

**Keywords:** timing of surgery, aSAH, time window, treatment modalities, prognosis

## Abstract

**Purpose:**

The objective of this study is to investigate the optimal timing of intervention therapy for aneurysmal subarachnoid hemorrhage (aSAH). This research aims to analyze the impact of early treatment and various intervention modalities on clinical outcomes in patients presenting with aSAH.

**Materials and methods:**

Patients with aSAH were analyzed for demographics, Fisher grading, WFNS grading, and Hunt-Hess grading. The modified Rankin Scale (mRS) compared outcomes between groups receiving clamping or embolization within 12 h vs. later. Logistic regression evaluated factors affecting mRS prognosis at 3 months post-discharge.

**Results:**

This study included 226 patients, with 192 (85%) receiving embolization and 34 (15%) patients underwent clipping. 58 (26%) treated within 12 h of aSAH onset and 168 patients received treatment after 12 h. No significant mRS differences were found between clamping and embolization. Predictors of length of hospitalization were predicted by age, Modified Fisher classification, WFNS classification, Hunt-Hess classification, complications, and intervention time under 12 h. Early intervention <12 h significantly affected the favorable outcome of mRS (0–2) after at 3 months post-discharge; higher admission score, advanced age, and the presence of complications were associated with poor prognosis.

**Conclusion:**

Early treatment within 12 h leads to improved clinical outcomes over delayed treatment.

## 1 Introduction

The global incidence of aneurysmal subarachnoid hemorrhage (aSAH) is 6.1/100,000,000 people (1). As the population ages, aSAH poses a growing threat to human life and health. Patients with aSAH have a high risk of rebleeding in the short term (2), and rapid occlusion of ruptured aneurysms is effective in decreasing the risk of rebleeding (3), and early surgical intervention can reduce the risk and improve the prognosis of patients (4). However, there is a notable discrepancy in defining early treatment for aSAH. The 2023 American Heart Association and Stroke Association Guidelines for the Management of Patients with aSAH recommends early occlusion of the bleeding aneurysm within 24 h (5), whereas the 2013 European Stroke Organization allows a 72-hour window (6). Therefore, the optimal surgical time window for patients with aSAH is still debated, lacking clear evidence. This study aims to refine this window by categorizing patients into different surgical time groups to identify an early treatment window that has a better prognosis for the patients through a more detailed time grouping.

## 2 Materials and methods

2.1 We retrospectively analyzed 226 patients with common aSAH who received endovascular spring coil embolization or surgical clamping at the Jining No.1 People's Hospital from 2020 to 2024. This does not include patients with complex aneurysms. This study was approved by the Medical Ethics Committee of Jining No.1 People's Hospital (No. JNRM-2021-DW-028). All of the procedures conducted were designed in strict accordance with the Declaration of Helsinki.

Inclusion criteria: SAH clearly caused by an intracranial-responsible aneurysm by CT angiography or digital subtraction angiography and underwent the corresponding surgical treatment.

Exclusion criteria: ① with subarachnoid hemorrhage but no intracranial responsible aneurysm was found after relevant examinations; ② exclusion of traumatic subarachnoid hemorrhage, subarachnoid hemorrhage caused by vascular malformations, and subarachnoid hemorrhage of unknown causes; ③ Patients who gave up the treatment or died due to aggravation of the condition without surgery; ④ Incomplete clinical data; ⑤ The history of aSAH or unruptured intracranial aneurysms treated with surgery; ⑥ Recent history of brain surgery, malignant tumors of other organs, and coagulation dysfunction; ⑦ Patients with complex aneurysms (complex cerebral aneurysms include those with irregular shapes, atypical locations, or those accompanied by other vascular abnormalities).

### 2.2 Clinical parameters

Patient baseline data included age, sex, and time from onset to undergoing surgery, World Federation of Neurological Surgeons (WFNS) Scale, Hunt-Hess classification, Modified Fisher Scale, aneurysm location, postoperative complications, and modified Rankin Scale (mRS) grade at 3-month follow-up after discharge. All the surgeries were performed by the same group of experienced surgeons, and all patients received the same perioperative management plan.

### 2.3 Assessment of outcomes

Clinical outcomes at 3-month follow-up after discharge were assessed with the modified Rankin rating Scale (mRS), defining 0–2 as better prognosis and 3–6 as worse prognosis group.

## 3 Statistical analysis

3.1 Statistical analyses were performed using SPSS version 21.0 (IBM SPSS Statistics 21). Firstly, the data of baseline information were tested for normal distribution, categorical variables were expressed as numbers and percentages, and measures were tested by the Shapiro–Wilk test. Those that conformed to a normal distribution were expressed as $\left(\bar{x}\pm s\right)$, and those that did not conform to a normal distribution were expressed as median and quartile. Quartiles M (P25, P75) were expressed.

3.2 Categorical variables were assessed for significance of differences between groups using Pearson's chi-square test or Fisher's exact test.

3.3 Variables with a correlation of *p* ≤ 0.05 in the univariate analysis were entered into a multivariate logistic regression model. Ordered multi-categorical logistic regression was used to analyze the risk factors for different prognostic outcomes of the patients with a test level of *α* = 0.05.

3.4 For groups with significant differences in size, propensity score matching and inverse probability weighting were performed. The propensity score was estimated using binary logistic regression, with the surgical group as the dependent variable and all baseline variables requiring balance as independent variables. Matching was conducted using the nearest neighbor method, with a caliper set at 0.1 times the standard deviation of the propensity score. Inverse probability weighting was calculated using binary logistic regression to generate weights, followed by a weighted logistic regression analysis.

## 4 Results

### 4.1 Comparison of severity of hospital admission in the 4 groups

**Figure 1:**
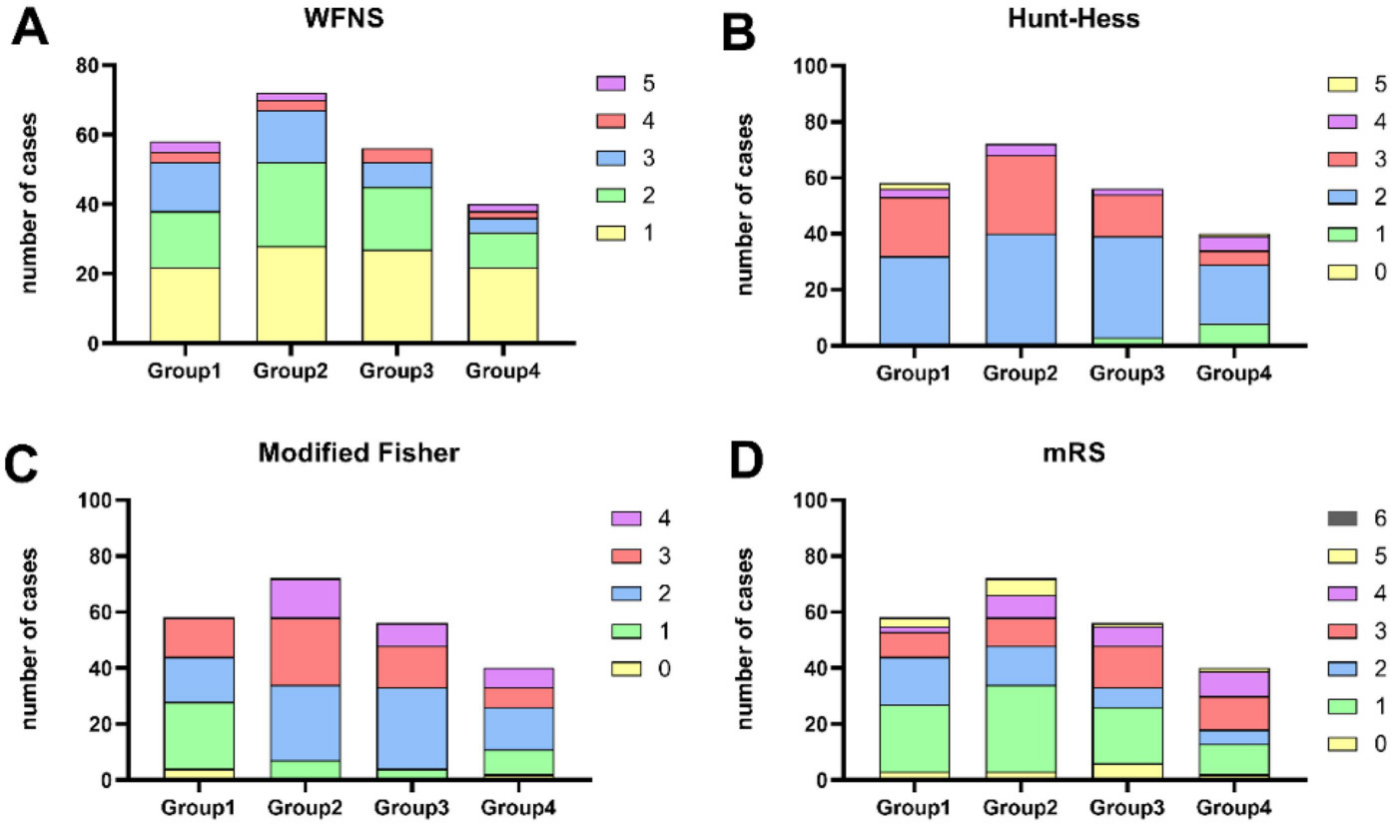
The distribution of various scores in different groups. WFNS score **(A)**, Hunt-Hess score **(B)**, Modified Fisher score **(C)**, mRS score **(D)**.

According to the neurological assessments conducted on the patient upon admission, namely the Hunt-Hess score, modified Fisher score, and WFNS score. For statistical convenience, patients with Hunt-Hess scores of 0–2, modified Fisher scores of 0–2, and WFNS scores of 1–3 were classified as mildly ill, while those with higher scores were deemed more severely ill (Table 1 and Figure 1A–C) it can be obtained that there were no significant differences in severity among the four groups at admission (*p* > 0.05).

**Table 1:** Clinical characteristics of patients.

	0–12 (*n* = 58)	12–24 (*n* = 72)	24–48 (*n* = 56)	>48 (*n* = 40)	*p* value
Age	59.43 ± 12.17	57.94 ± 11.42	59.38 ± 11.44	61.2 ± 7.75	0.521
Gender					0.803
Male	18 (31%)	19 (26%)	14 (25%)	13 (33%)	
Female	40 (69%)	53 (74%)	42 (75%)	27 (67%)	
WFNS					0.865
Grade 1–3	52 (90%)	67 (79%))	52 (93%)	36 (90%)	
Grade 4–5	6 (10%)	5 (21%)	4 (7%)	4 (10%)	
Hunt-Hess					0.141
Grade 0–2	32 (55%)	40 (56%)	39 (70%)	29 (73%)	
Grade 3–5	26 (45%)	32 (44%)	17 (30%)	11 (27%)	
Modified Fisher					0.206
Grade 0–2	44 (76%)	34 (47%)	33 (59%)	26 (65%)	
Grade 3–4	14 (24%)	38 (53%)	23 (41%)	14 (35%)	
Location					0.326
Anterior circulation	30 (52%)	42 (58%)	29 (52%)	16 (40%)	
Posterior circulation	28 (48%)	30 (42%)	27 (48%)	24 (60%)	
Complication	16 (28%)	11 (15%)	13 (23%)	12 (30%)	0.239

WFNS, World Federation of Neurological Surgeons score; Complications include delayed cerebral ischemia, cerebral infarction, hydrocephalus, intraventricular hemorrhage, rebleeding, and symptomatic seizures. The anterior circulation consists of the anterior cerebral artery, the middle cerebral artery and the anterior communicating artery; the posterior circulation includes the vertebral artery, the basilar artery and their branches.

### 4.2 The prognosis of patients with different surgical timing and surgical methods

#### 4.2.1 Comparison of the prognosis of patients in the 2 groups with different surgical procedures

Patients were divided into clamping and embolization groups based on their surgical methods. Three months after discharge, an mRS score of 0–2 is considered a better prognosis, while a score of 3–6 indicates a poorer prognosis. Comparison of patients who underwent different surgical procedures did not show any significant difference in discharge prognosis (Table 2, *p* > 0.05), which led to the conclusion that surgical procedure was not an independent factor affecting patients' prognosis.

**Table 2:** Clinical outcomes in patients with aneurysmal subarachnoid hemorrhage.

	Embolization (*n* = 192)	Clipping (*n* = 34)	*p*	Group 1	Group 2	Group 3	Group 4	*p*
mRS			0.175					0.015
0–2	125 (65%)	18 (53%)		44 (76%)	48 (67%)	33 (59%)	18 (45%)	
3–6	67 (35%)	16 (47%)		14 (34%)	24 (33%)	23 (41%)	22 (55%)	

mRS, modified Rankin Scale.

Furthermore, due to the substantial difference in the number of patients between the two groups, we conducted refined propensity score matching (PSM) and inverse probability weighting. As shown in Table 3, there were no statistically significant differences in gender, age, severity of illness at admission, complications, or 3-month post-discharge prognosis scores between the embolization and clipping groups. Only the aneurysm location demonstrated a statistically significant difference (*p* < 0.05). Subsequently, after performing 1:1 PSM based on several confounding factors, no significant difference in prognosis remained between the two groups (Table 4, *p* > 0.05).

**Table 3:** Baseline data of aSAH patients with different treatment.

	Embolization (*n* = 192)	Clipping (*n* = 34)	*p* value
Gender			0.570
Male	53	11	
Female	139	23	
Age	59.08 ± 11.41	60.26 ± 8.91	0.565
WFNS			0.924
Grade 1–3	176	31	
Grade 4–5	16	3	
Hunt-Hess			0.429
Grade 0–2	121	19	
Grade 3–5	71	15	
Modified Fisher			0.540
Grade 0–2	118	19	
Grade 3–4	74	15	
mRS			0.175
Grade 0–2	125	18	
Grade 3–6	67	16	
Location			0.040
Anterior circulation	94	23	
Posterior circulation	98	11	
Complication	46	6	0.420

WFNS, World Federation of Neurological Surgeons score; Complications include delayed cerebral ischemia, cerebral infarction, hydrocephalus, intraventricular hemorrhage, rebleeding, and symptomatic seizures.

**Table 4:** Baseline data of patients with different treatments methods after PSM.

	Embolization (*n* = 33)	Clipping (*n* = 33)	*p* value
Gender			0.592
Male	9	11	
Female	24	22	
Age	63.00 ± 9.85	60.52 ± 8.93	0.287
Location			0.306
Anterior circulation	19	23	
Posterior circulation	14	10	

To further validate this conclusion, we applied inverse probability weighting to patients in both groups and developed a binary logistic regression model. As shown in Table 5, within the population stratified by surgical method, age, gender, severity of illness at admission, aneurysm location, and presence of complications all significantly influenced patient prognosis (*p* < 0.05), whereas the surgical method did not have a statistically significant effect. In conclusion, within this study population, the surgical method is not an independent factor affecting patient prognosis.

**Table 5:** Logistic regression analysis using inverse probability weighting.

Variable	Coefficient	ORs	95% CI	*p* value
Age	0.031	1.031	1.021–1.041	<0.001
Gender	0.552	1.737	1.333–2.263	<0.001
Hunt-Hess	1.128	3.090	2.386–4.002	<0.001
WFNS	2.131	8.422	5.079–13.965	<0.001
Modified Fisher	1.047	2.849	2.245–3.616	<0.001
Location	0.403	1.496	1.150–1.946	0.003
Treatment	0.434	1.543	0.749–3.178	0.239
Complication	0.725	2.065	1.636–2.606	<0.001

#### 4.2.2 Comparison of the prognosis of patients with different timing of surgery in the 4 groups

**Figure 2:**
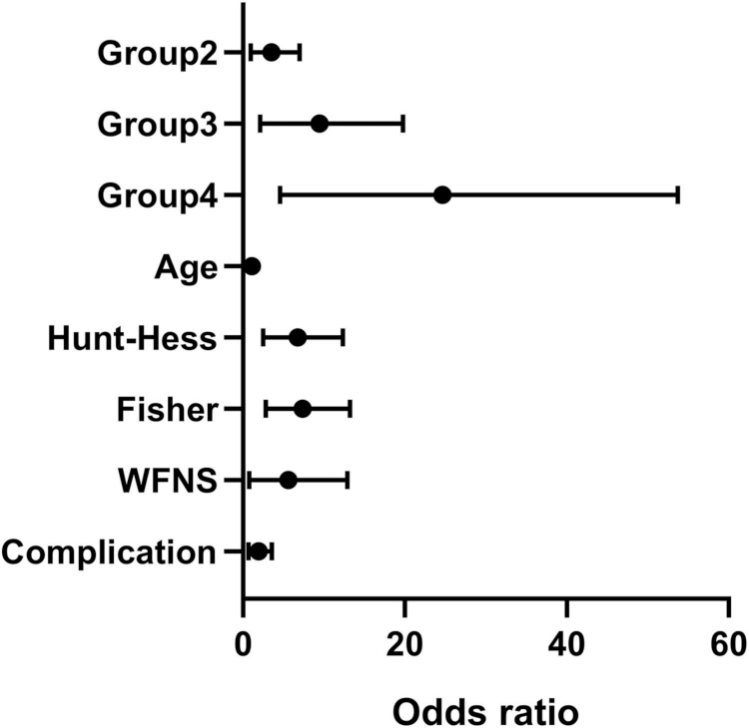
Forest plots of factors associated with prognosis.

Furthermore, there was a statistically significant difference in prognostic outcomes based on surgical timing (*p* < 0.05), which allowed for the development of constructing a model with unifactorial and multifactorial logistic regression analyses (Tables 2, 6 and Figures 1D, 2).

**Table 6:** Logistic regression analysis to assess the factors associated with outcome in the modified Rankin scale.

Variable	Coefficient	ORs	95% CI	*p* value
Group				
2	0.945	2.574	0.945–7.011	0.064
3	1.868	6.478	2.125–19.479	0.001
4	2.751	15.661	4.566–53.717	0.000
Age	0.069	1.071	1.035–1.109	0.000
Hunt-Hess	1.709	5.524	2.478–12.311	0.000
Fisher	1.808	6.098	2.814–13.214	0.000
WFNS	1.138	3.119	0.754–12.911	0.116
Complication	0.425	1.530	0.659–3.554	0.323

WFNS, World Federation of Neurological Surgeons score; Complications include delayed cerebral ischemia, cerebral infarction, hydrocephalus, intraventricular hemorrhage, rebleeding, and symptomatic seizures.

### 4.3 Logistic analysis of prognostic-related factors using mRS scores

The baseline data of patients admitted to the hospital were sequentially subjected to univariate analysis, and those with *p* < 0.05 were entered into multifactorial logistic regression analysis, and ordered multicategorical logistic regression was used to analyze the risk factors for different prognostic outcomes of the patients, with a test level of *α* = 0.05. Group 1 was set as a dummy variable, and Groups 2, 3, and 4 were analyzed with Group 1 as the reference group, respectively. As shown in Table 3, the probability of a worse prognosis in Group 2 was 2.57 times higher than in Group 1, while Group 3 and 4 had probabilities 6.47 and 15.6 times higher, respectively. In addition, age, admission Hunt-Hess classification, higher modified Fisher classification and WFNS score, and complications were also risk factors for poor outcomes.

## 5 Discussion

Aneurysmal subarachnoid hemorrhage (aSAH) is a complex cerebrovascular disease that is prone to severe complications and high mortality. It represents 5% of all strokes, with a global incidence of about 700,000 cases annually and a mortality rate of approximately 40% (7, 8). The main treatment for aSAH is surgical, typically involving vascular clamping or interventional embolization (9). Patients should be admitted to the hospital promptly after symptom onset to assess their condition, identify the aneurysm, and begin surgical treatment, thereby delaying disease progression and minimizing complications (10).

Timely surgery is essential for improving recovery prognosis. Early intervention can effectively remove hematomas before tissue edema, preventing primary effects and secondary injuries (11). However, there is no consensus on the optimal timing for surgery after onset, and most studies have long time spans. This study analyzed 226 patients, collecting clinical data and creating detailed time groupings to define a precise treatment window for early surgery. Our study included patients with surgery times from 3 h to 8 days. We primarily grouped those treated within 48 h, finding that surgery within 12 h of onset led to a better prognosis. An intracranial hematoma typically forms 30 min after an aneurysm rupture, peaking in 1–2 h. Patients can gradually control the bleeding phenomenon under the action of their own coagulation system, and this process is generally within 12 h, stabilizing their condition. This allows for safer early surgery to prevent secondary brain injury, limit hematoma expansion, reduce the risk of the hematoma, and reduce the chances of forming a cerebral herniation. Therefore, it can be considered that the best time for surgical treatment is before edema forms around the hematoma, minimizing damage to surrounding brain tissue (12, 13). If left uncontrolled, the hematoma can expand, worsening the condition and causing secondary brain damage, disrupting the blood-brain barrier, and increasing cytotoxicity from cell lysis products (14). However, some studies have suggested that patients admitted early still face severe bleeding, and surgery at this time would not only increase the difficulty of surgery but also affect the function of the fibrinolytic-coagulation system, increasing surgical risks (14, 15).

A study by Zhao et al. with 118 patients, complications and outcomes were similar in the ultra-early surgery group vs. the delayed surgery group, with no significant differences (16). This seems to indicate that ultra-early surgery may not provide the expected benefits. However, the 6–12 h window after aSAH has a high risk of rebleeding. Park et al. found that patients with low-grade aSAH who had surgery within 24 h had better outcomes than those who had surgery after 3 days, with reduced rebleeding risk due to timely aneurysm localization, clamping, or mobilization. However, in patients with high-grade aSAH patients, surgery timing did not significantly impact outcomes (17). This may be due to the fact that high-grade patients within this study were admitted with an irreversible state, and early or late surgery could have improved their condition only to a limited extent. Therefore, we need a study with a larger sample size and a finer time window. Another meta-analysis by Wei et al. mentioned (18) that the emergence of deferred surgery was limited by the technology available at the time, and now, more surgeons believe that early or even ultra-early surgery is more likely to reduce the risk of complications and death in their patients, and the findings of our study are consistent with this.

While Tomasz Tykocki's 2017 study focused on low-grade aSAH patients with a small sample size (19), our study included fully graded patients, not just low-grade aSAH cases, enhancing the rigor of our grouping. This accurate classification clarifies prognostic factors for high-grade patients and informs future treatment strategies and optimal surgical timing.

Our study has limitations, including its retrospective nature and a small number of complication cases per group, so we counted the presence or absence of complications, which may introduce bias. Meanwhile, we have not completely ruled out the confounding factors before the onset of the disease in the patients, such as the use of antiplatelet drugs. Existing studies have shown that the application of such drugs can lead to a poor prognosis for the patients (20). This suggests that future studies with larger sample sizes should more comprehensively consider the factors influencing disease onset in patients. As a single-center study, future research with multiple centers and larger sample sizes is necessary to overcome these shortcomings.

## 6 Conclusions

Our study showed that patients with aSAH who underwent surgery at 12 h from onset had a better prognosis than those treated for a longer period. The results also proved that there was no significant difference in prognosis between the two mainstream surgical methods.

## Data Availability

The original contributions presented in the study are included in the article/Supplementary Material, further inquiries can be directed to the corresponding authors.
